# Functional Characterization of Trypsin in the Induction of Biologically Live Bait Feeding in Mandarin Fish (*Siniperca chuatsi*) Larvae

**DOI:** 10.3390/cells14191537

**Published:** 2025-10-01

**Authors:** Xiaoru Dong, Ke Lu, Jiaqi Wu, Qiuling Wang, Xu-fang Liang

**Affiliations:** 1 College of Fisheries, Chinese Perch Research Center, Huazhong Agricultural University, Wuhan 430070, China; xiaorudong@webmail.hzau.edu.cn (X.D.); luke@webmail.hzau.edu.cn (K.L.); 2021308010016@webmail.hzau.edu.cn (J.W.); qiulingwang_230714@webmail.hzau.edu.cn (Q.W.); 2 Engineering Research Center of Green Development for Conventional Aquatic Biological Industry in the Yangtze River Economic Belt, Ministry of Education, Wuhan 430070, China

**Keywords:** mandarin fish (*Siniperca chuatsi*), trypsin, digestive, appetite, expression

## Abstract

The early developmental transition from endogenous to exogenous feeding is a critical period in carnivorous fish larvae, often associated with high mortality rates in aquaculture. Although trypsin, a key protease in protein digestion, is hypothesized to play a pivotal role in initiating exogenous feeding, the expression dynamics and functional contributions of trypsin and isoforms during early development remain poorly characterized in carnivorous species. This study explores the critical role of trypsin in the early feeding process of carnivorous fish, using mandarin fish (*Siniperca chuatsi*) as a model, which is a commercially valuable species that faces significant challenges during this phase due to its strict dependence on live prey and underdeveloped digestive system. Phylogenetic analysis indicates that, compared to herbivorous and omnivorous fish, carnivorous fish have evolved a greater number of trypsins, with a distinct branch specifically dedicated to try. RNA-seq data revealed the expression profiles of 13 trypsins during the early developmental stages of the mandarin fish. Most trypsins began to be expressed in large quantities with the appearance of the pancreas, reaching a peak prior to feeding. In situ hybridization revealed the spatiotemporal expression pattern of trypsins, starting from the pancreas in early development and later extending to the intestines. Furthermore, inhibition of trypsins activity successfully suppressed early oral feeding in mandarin fish, which was achieved by increasing the expression of cholecystokinin 2 (CCK2) and proopiomelanocortin (POMC) to suppress appetite. These findings enhance our understanding of the adaptive relationship between the ontogeny of the digestive enzyme system and feeding behavior in carnivorous fish. This research may help alleviate bottleneck issues in aquaculture production by improving the survival rate and growth performance of carnivorous fish during critical early life stages.

## 1. Introduction

The feeding habits of carnivorous fish are closely aligned with their digestive enzyme systems, both structurally and functionally, to support the efficient breakdown of high-protein diets. For example, albacore tuna (*Thunnus alalunga*) rapidly degrades prey muscle proteins through highly active trypsin and chymotrypsin, facilitating efficient digestion after ingesting small fish or cephalopods [1]. Similarly, both largemouth bass (*Micropterus salmoides*) and north pacific bluefin tuna (*Thunnus orientalis*) rely on potent gastric pepsins to break down high-protein prey, adapting to their strongly carnivorous feeding habits [2,3]. During early development, the digestive systems of carnivorous fish undergo significant differentiation, with shifts in digestive enzyme composition and activity [4,5,6]. Notably, many carnivorous fish lack a functional stomach with gastric glands at larval stages, relying instead on pancreatic proteases—especially trypsin—for protein digestion [7,8]. The pancreas synthesizes alkaline proteases, with trypsin being the predominant and most abundant protease during the early feeding stages of larvae [9,10]. Trypsin, a key serine protease, not only hydrolyzes dietary proteins but also activates other proteolytic enzymes through cascade mechanisms, playing a central role in digestion and other physiological processes [11,12].

The transition from endogenous yolk nutrition to exogenous feeding is a critical period in larval development. The activation of digestive enzymes, such as trypsin, often precedes the initiation of feeding behavior, indicating that its regulatory mechanism is genetically programmed rather than solely induced by dietary signals [7]. This premature activation serves as a critical preparatory adaptation, ensuring a successful transition in nutritional sources for the larvae. Furthermore, the emergence and activation of trypsin represent a means of triggering intracellular signaling pathways, which can regulate gene expression, cell proliferation, differentiation, and inflammatory responses [13]. The regulation of trypsin involves multiple factors, including appetite-related hormones. For instance, knockout of the appetite-suppressing receptor npy8br in zebrafish reduces trypsinogen expression, while cholecystokinin (CCK) exerts a negative feedback effect on trypsinogen [14,15,16,17]. Neuropeptide Y (NPY) promotes the expression of trypsin and amylase in grass carp (*Ctenopharyngodon idella*) [18]. Conversely, trypsin also influences the expression of appetite factors, and inhibition of trypsin activity similarly leads to reduced appetite, potentially involving the regulation of CCK-related pathways [19]. This bidirectional interaction between trypsin and appetite factors adds complexity to the functional role of trypsin.

As an economically important and typical carnivorous freshwater fish species, the mandarin fish (*Siniperca chuatsi*) farming industry has developed rapidly in recent years. The aquaculture yield reached 400,000 metric tons in 2022, making it one of the pillar industries of Chinese aquaculture [20]. Newly hatched mandarin fish larvae have underdeveloped digestive organs: the intestine exhibits only primary folds, pancreatic tissue differentiates into discrete cell clusters, and the stomach remains in a primitive morphological state [21]. This unique biological phenomenon suggests that mandarin fish larvae heavily rely on highly efficient trypsin expression to digest live prey [22,23]. However, the mechanisms by which trypsin retroactively regulates appetite factors and influences the feeding behavior of mandarin fish remain to be elucidated.

In this study, we analyzed and compared the expression profiles of all trypsin genes during the early developmental stages of mandarin fish. Key subtype genes were selected, and their expression patterns and locations during the early developmental stages of mandarin fish larvae were determined. Furthermore, the effect of trypsin on the initiation of exogenous feeding in mandarin fish larvae was explored through the use of trypsin inhibitors. This study aims to elucidate the adaptive relationship between the digestive enzyme system and feeding behavior in carnivorous fish species like mandarin fish. The findings will not only deepen the theoretical understanding of evolutionary digestive physiology in fish but also provide insights into improving the initial feeding success of mandarin fish larvae by stimulating trypsin expression.

## 2. Materials and Methods

### 2.1. Fish Farming and Sample Collection

The broodstock used in this study were provided by the Chinese perch research center at Huazhong Agricultural University (Wuhan, China) for standardized breeding. During the farming process, environmental parameters were maintained as follows: dissolved oxygen in water at 7–8 mg/L, water temperature at 25.0 ± 1.0 °C, and pH between 7.8 and 8.3. After artificial fertilization, fertilized eggs were collected and placed in a 25 °C constant temperature incubation system for embryo development monitoring. Five consecutive developmental time points (24, 48, 72, 96, and 120 h post-fertilization, hpf) were selected for sample collection during the period from 24 to 120 hpf for in situ hybridization and RT-qPCR analyses. Samples intended for in situ hybridization were fixed in 4% paraformaldehyde (PFA, Beyotime Biotechnology, Shanghai, China, P0099) at 4 °C for 24 h, whereas samples for RT-qPCR were flash-frozen in liquid nitrogen and stored at −80 °C. Prior to sampling, all embryos/larvae were deeply anesthetized using tricaine methanesulfonate (MS-222, 100 mg/L; Sigma-Aldrich, St. Louis, MO, USA). At 120 hpf (mandarin fish initiated feeding behavior), two subgroups were further established: a fasting group and a live prey-feeding group (fed with zebrafish larvae, *Danio rerio*). Samples were collected after deep anesthesia with MS-222 (100 mg/L). Once the individuals lost their stress responses, they were fixed in 4% PFA (for in situ hybridization) or rapidly frozen in liquid nitrogen, then stored at −80 °C for long-term preservation (for mRNA extraction). This study was conducted in strict accordance with the “Laboratory Animal Guidelines” by the Ministry of Science and Technology of China (Beijing, China) and was approved by the Animal Experiment Ethics Committee of Huazhong Agricultural University (Approval No. HZAUFI-2025-0037, Wuhan, Hubei). All experimental designs adhered to the principles of animal welfare, and the sampling procedures were optimized to minimize pain and distress to the experimental fish.

### 2.2. The mRNA Expression of Trypsin in Mandarin Fish

RNA-Seq data from different developmental stages of healthy mandarin fish larvae were retrieved from the NCBI SRA database (https://www.ncbi.nlm.nih.gov/; 12 January 2025), including samples at 0 hpf (SRR15291860, SRR15291871, SRR15291872), 12 hpf (SRR15291866, SRR15291867, SRR15291868), 24 hpf (SRR15291859, SRR15291861, SRR15291862), 48 hpf (SRR15291846, SRR15291847, SRR15291848), 69 hpf (SRR15291843, SRR15291844, SRR15291845), 96 hpf (SRR8109010), 120 hpf (PRJNA1051490), and 192 hpf (SRR8109009), covering a total of 20 tissue transcriptomes [24]. Gene expression quantification was performed on all RNA-seq data, and samples were normalized according to TPM (transcripts per kilobase million) [25]. A heatmap of trypsin gene expression was generated using GraphPad Prism software (version 9.0).

### 2.3. Sequence and Phylogenetic Analysis

To elucidate phylogenetic relationships of trypsin genes in mandarin fish, we collected 73 trypsin genes from 8 species, including mammals (*Homo sapiens* and *Mus musculus*), 2 carnivorous fish species (*S. chuatsi* and *Esox lucius*), 2 herbivorous fish species (*Ctenopharyngodon idella* and *Megalobrama amblycephala*), and 2 omnivorous fish species (*Danio rerio* and *Oryzias latipes*) (Supplementary Table S1). The amino acid sequences were aligned using MEGA X, and a phylogenetic tree was constructed using Maximum Likelihood (ML) method with 1000 bootstrap iterations, with all other parameters set to default values. The phylogenetic tree was then visualized and edited using ITOL (v7, https://itol.embl.de/). The protein tertiary structures of trypsin genes from humans and mandarin fish were predicted using AlphaFold 3 (https://alphafold.ebi.ac.uk/; 15 February 2025). Statistical differences were analyzed in GraphPad Prism software (version 9.0).

### 2.4. Whole-Mount in Situ Hybridization

As described in previous studies [26,27], the mRNA expression patterns were detected using the whole-mount in situ hybridization (WISH) technique. In all WISH experiments, embryos and larvae were fixed in 4% PFA (4 °C, 12 h) and then subjected to sequential washes in PBST (containing 0.1% Tween-20), followed by gradient dehydration in PBST-methanol (25–50–75%). Finally, they were stored in methanol at −20 °C. The DIG RNA Labeling Kit (SP6/T7) (Roche, Mannheim, Germany, 11175025910) was used to transcribe digoxigenin-labeled TRY antisense ribonucleotide probes in vitro, following the manufacturer’s instructions. Anti-Digoxigenin-AP, Fab fragments (Roche, 11093274910) were used to bind the probes, and the NBT/BCIP color development system (Roche, 72091) was applied for signal visualization. A sense probe was used as a control to avoid non-specific binding. The spatial expression patterns were recorded using a stereomicroscope (Olympus SZX16). The primers used for whole-mount in situ hybridization are listed in Supplementary Table S2.

### 2.5. Trypsin Functional Targeted Inhibition Assay

The trypsin inhibitor (TI, Biosharp Biotechnology, Catalog No. BS158, Beijing, China) was prepared as a stock solution at a concentration of 20 g/L and stored in aliquots at 4 °C in a light-protected container. On the day of the experiment, the stock solution was diluted in rearing water to prepare four treatment groups with final concentrations of 0.02, 0.2, 2, and 20 mg/L, with an equal volume of rearing water serving as the control group. A total of 100 healthy larvae (4.744 ± 0.149 mm) were selected for each group and placed into 1 L of the respective treatment solution, exposed for 2 h in an incubator maintained at 25 °C. The larvae were fed live prey fish (zebrafish) at a ratio of 1:1.5, and their feeding proportion (the percentage of fish that were feeding in each group) was recorded over the 2 h exposure period for each group, with four repetitions. Given the potential of trypsin inhibitor (TI) to suppress chymotrypsin, we first examined the early expression of chymotrypsin in mandarin fish larvae and further assessed its expression following TI inhibition.

### 2.6. Determination of Digestive Enzyme Activity

After 2 h of TI immersion, samples were collected from five groups of mandarin fish (0.02, 0.2, 2, 20 mg/L TI and control), with 50 individuals per group and four repetitions [28]. This study aimed to investigate the effects of different treatments on trypsin activity in fish. All litters were anesthetized with 100 mg/L MS-222 and then sampled and stored at −80 °C. For digestive enzyme extraction, the samples were first homogenized with 10 times the volume of saline and then centrifuged at 4000× *g* for 10 min at 4 °C. After centrifugation, the supernatant was collected for enzyme activity determination. Then, the total protein content (Kemin Biologicals, KMSP-1-W, Suzhou, China), trypsin activity (Kemin Biologicals, YPT-1-W, Suzhou, China), were determined according to the instructions. The protein content was expressed as mg/mL, and trypsin activity was expressed as U/mg prot.

### 2.7. The qRT-PCR and Statistical Analysis

Total RNA from the samples was extracted using the RNAiso Plus Kit (Takara, Beijing, China, Cat. No. 9109). cDNA synthesis was performed using the PrimeScript RT Reagent Kit with gDNA Eraser (TaKaRa, Cat. No. RR047A). Gene expression analysis for each sample was conducted using the TB Green^®^ Premix Ex Taq™ II (Tli RNaseH Plus) Kit (TaKaRa, Cat. No. RR820A) on the CFX96 Touch™ Real-Time PCR Detection System (Bio-Rad, Hercules, CA, USA). The relative expression levels of genes were compared across different samples using the 2^−ΔΔCt^ method [29]. Statistical differences between groups were analyzed using one-way analysis of variance (ANOVA) in GraphPad Prism software (version 9.0). A significance threshold was set at *p* < 0.05 (significant) and *p* < 0.01 (highly significant). All experimental data included three independent biological replicates, and results are presented as mean ± standard error. The primers used for RT-qPCR are listed in Supplementary Table S3.

## 3. Results

### 3.1. Phylogenetic Analysis of Trypsins

**Figure 1:**
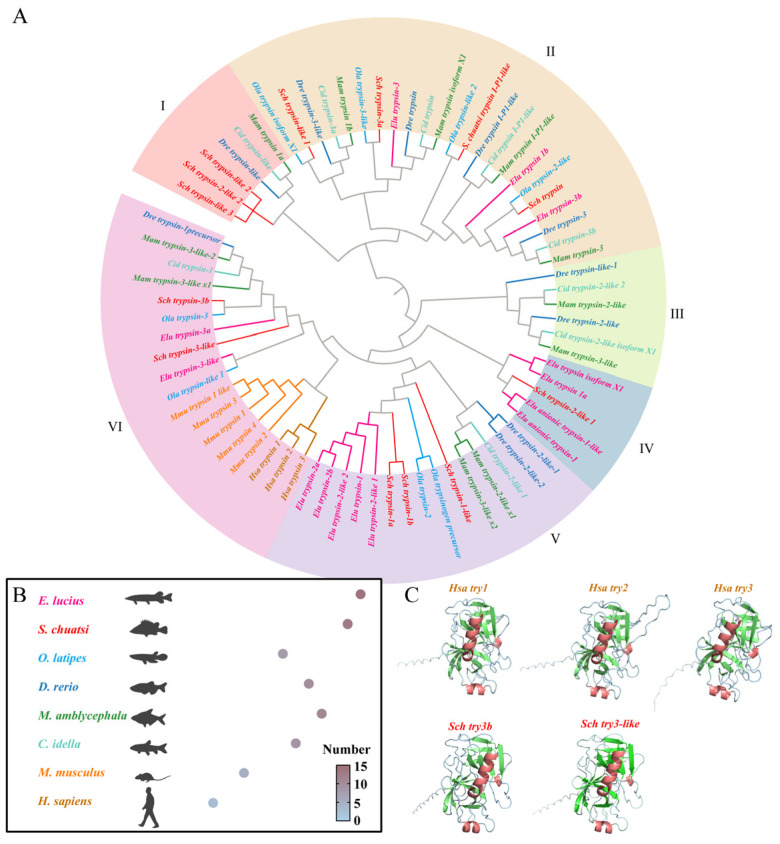
Phylogenetic analysis and protein tertiary structure prediction of trypsin genes. (**A**) Phylogenetic tree of 8 species. (**B**) Gene count of trypsin genes in 8 species. (**C**) Tertiary structure prediction of human trypsins and mandarin fish *trypsin-3b* and *trypsin-3-like* genes, with red representing α-helix, green representing β-sheet, and blue representing random coil.

To gain deeper insights into the evolution of trypsin in teleost fishes, we identified and collected 73 trypsins from 8 species, including two mammals (*H. sapiens* and *M. musculus*), two carnivorous fishes (*S. chuatsi* and *E. lucius*), two herbivorous fishes (*C. idella* and *M. amblycephala*), and two omnivorous fishes (*D. rerio* and *O. latipes*) and constructed a phylogenetic tree (Figure 1A). The results indicate that, compared to mammals, teleosts have undergone significant expansion of trypsins, with carnivorous fish species having a higher number of trypsins than omnivorous and herbivorous fish species (Figure 1A,B). Furthermore, based on the clustering of the phylogenetic tree, we classified trypsins into six classes. While Class III is absent in carnivorous fish species, Class IV is exclusively restricted to this group. Additionally, two trypsins from mandarin fish (trypsin-3-like and trypsin-3b) cluster together with human and mouse trypsins, suggesting that they may be orthologous genes (Figure 1A). Comparative structural predictions of human trypsins and mandarin fish trypsin-3-like and trypsin-3b genes demonstrated a high degree of similarity in their tertiary protein configurations (Figure 1C).

### 3.2. Expression Patterns of Trypsins During Different Developmental Stages in Mandarin Fish

**Figure 2:**
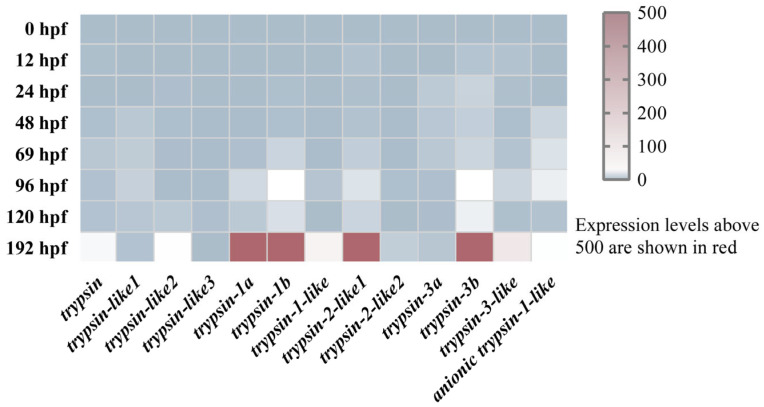
Heatmap of the expression of 13 trypsin gene members in mandarin fish during the 0–192 hpf developmental stages. The color gradient represents the expression level of TPM, with the scale on the right indicating the range of raw TPM values (0–500). TPM values exceeding 500 are represented in red-brown.

To explore the expression patterns of trypsins during different developmental stages in mandarin fish larvae, we identified 13 trypsins in mandarin fish genome. Using RNA-Seq data, we analyzed the expression profiles of trypsins at eight developmental stages (0, 12, 24, 48, 69, 96, 120, and 192 hpf). The results indicated that from 0 to 69 hpf, the expression levels of all trypsins were very low (with TPM values below 3). At 96 hpf, the expression of two genes, trypsin-1b and trypsin-3b, increased, with TPM values of 6.25 and 6.38, respectively. By 192 hpf, 10 genes exhibited TPM values greater than 10, with four genes, including *trypsin-1a*, *trypsin-1b*, *trypsin-2-like1*, and *trypsin-3b*, showing TPM values over 500 (Figure 2). Similarly, we examined the early expression of pepsin, amylase, and chymotrypsin in mandarin fish larvae. The results revealed that all three enzymes were either expressed at extremely low levels or were undetectable in the larvae from 24 to 120 hpf (Figure S1).

### 3.3. Expression Localization of Trypsin-3b During the Development of Mandarin Fish Larvae

**Figure 3:**
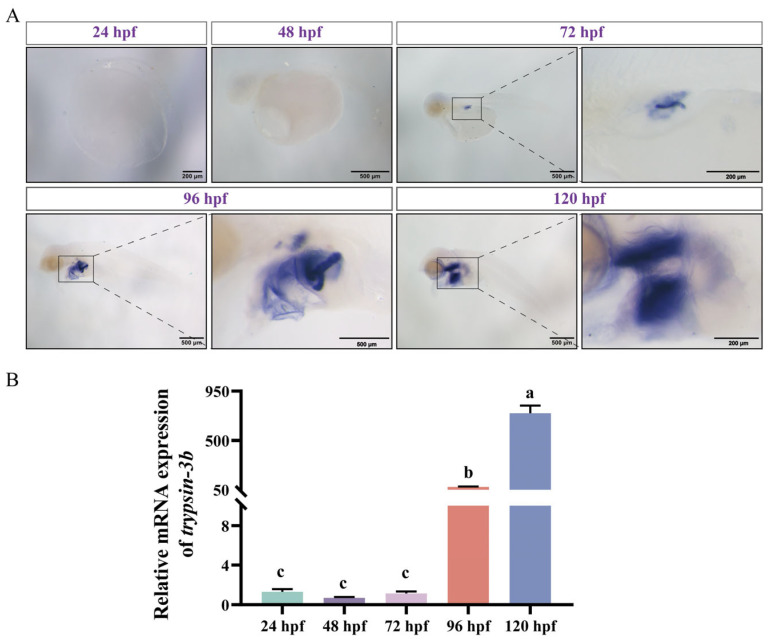
Spatiotemporal expression pattern of *trypsin-3b* in mandarin fish. (**A**) whole-mount in situ hybridization (WISH) of *trypsin-3b* in mandarin fish at 24, 48, 72, 96, and 120 hpf. The purple area denotes sites of hybridization of probe. (**B**) RT-qPCR quantification analysis of *trypsin-3b* expression in mandarin fish at 24, 48, 72, 96, and 120 hpf.

**Figure 4:**
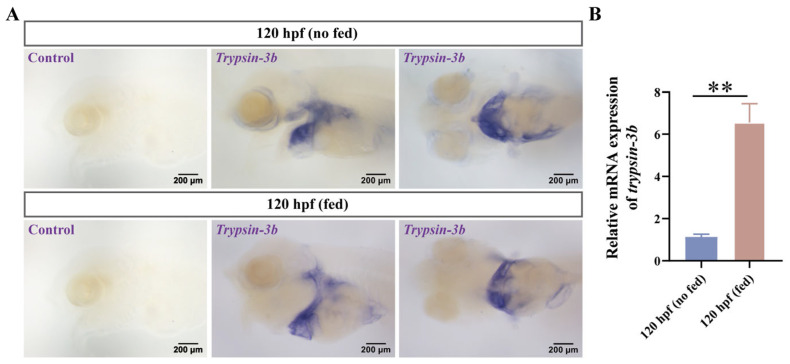
Expression pattern of *trypsin-3b* before and after first mouth opening in mandarin fish. (**A**) WISH of *trypsin-3b* in mandarin fish embryos before and after the first mouth opening (120 hpf), with purple indicating hybridization sites. (**B**) RT-qPCR quantification analysis of *trypsin-3b* expression before and after the first mouth opening (120 hpf) in mandarin fish. ** indicates *p* < 0.01.

To further elucidate the spatial and temporal expression dynamics of trypsin during development, we selected *trypsin-3b* as a representative gene and examined its expression pattern during early development of mandarin fish. In situ hybridization revealed that *trypsin-3b* expression was barely detectable at 24 and 48 h post-fertilization (hpf). By 72 hpf, weak expression was observed at the base of the abdominal region. Prominent expression was detected in the pancreas at 96 hpf, and by 120 hpf, strong expression was evident in both the intestine and pancreas, with a marked increase in signal intensity (Figure 3A). We further validated trypsin expression using RT-qPCR, which confirmed a significant upregulation at 96 hpf and a further increase at 120 hpf, showing a statistically significant difference compared to the expression level at 96 hpf (Figure 3B). We also compared the expression differences between mandarin fish larvae that had not yet started feeding and those that began feeding at 120 hpf. The expression regions of *trypsin-3b* remained unchanged before and after feeding, with abundant expression in the intestine and abdomen. However, after the larvae began feeding, there was a significant increase in expression, and the intensity of expression was stronger (Figure 4A). The RT-qPCR results were consistent with this trend (Figure 4B).

### 3.4. Effects of Trypsin Inhibitor on Feeding and Digestion in Mandarin Fish Larvae

**Figure 5:**
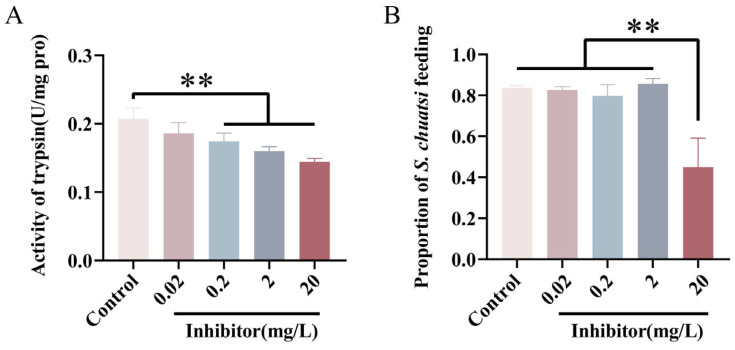
Inhibition of trypsin activity reduces the feeding rate of mandarin fish during mouth-opening stage. (**A**) Trypsin activity in mandarin fish is significantly inhibited with increasing inhibitor concentrations (0.02, 0.2, 2, 20 mg/L). (**B**) High inhibitor concentration (20 mg/L) leads to a decrease in the feeding rate during the first mouth-opening stage in mandarin fish. ** indicates *p* < 0.01.

To further investigate the function of trypsin, we exposed mandarin fish larvae to four concentrations of trypsin inhibitor (TI) at 0.02, 0.2, 2, and 20 mg/L. The results showed that, compared to untreated group, trypsin activity in the larvae was significantly reduced at 0.2, 2, and 20 mg/L TI concentrations (Figure 5A). Although the expression level of chymotrypsin was extremely low, we measured its expression under treatment with the highest concentration (20 mg/L) of the trypsin inhibitor to rule out potential interference. The results showed no significant difference in chymotrypsin expression between the treatment group and the control group (Figure S2). Additionally, when observing the feeding behavior of mandarin fish larvae, we found that the feeding rate was significantly decreased at the 20 mg/L concentration of TI (Figure 5B).

### 3.5. Trypsin Exerts Its Appetite-Suppressing Effects Through Anorexigenic Factors CCK and POMC

**Figure 6:**
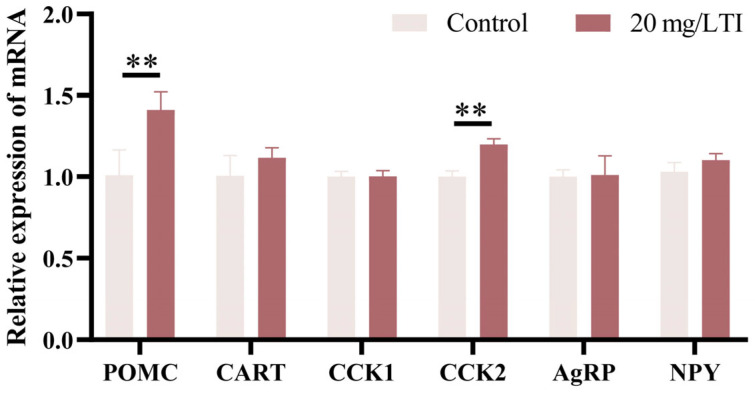
Inhibition of 20 mg/L TI upregulates the expression of appetite-regulating factors in mandarin fish. POMC, CART, and CCK are appetite-suppressing factors, and NPY and AGRP are appetite-stimulating factors. ** indicates *p* < 0.01.

To explore the cause of reduced feeding appetite in mandarin fish larvae after inhibition of trypsin activity, we analyzed the expression of appetite-related factors. Compared to untreated group, the inhibitor-treated group showed a significant increase in the expression of appetite-suppressing factors CCK and POMC. However, the expression of appetite-stimulating factors (NPY, and AgRP) did not exhibit significant changes. These results suggest that the inhibition of trypsin activity affects the expression of CCK and POMC, thereby suppressing feeding appetite in mandarin fish larvae (Figure 6 and Figure S3).

## 4. Discussion

As the most ancient and diverse group of vertebrates, the digestive system evolution pattern of fish has always been a key focus in the study of feeding adaptation and evolutionary patterns [30]. The mandarin fish (*S. chuatsi*), a highly valuable carnivorous fish species in Chinese freshwater aquaculture, faces a major challenge in its breeding stage due to the strict dependence of its fry on live prey during mouth-opening period, resulting in high mortality rates and severely restricting the development of large-scale farming [23]. This study revealed a significant expansion of the trypsinogen gene family in carnivorous fish genomes. We analyzed the expression dynamics and spatial distribution of trypsin during the early development of mandarin fish. Furthermore, inhibitor-based functional interference experiments demonstrated that trypsin regulates the mouth-opening feeding behavior of fish larvae. These findings suggest that trypsin may exert its function by inversely regulating the expression of appetite-suppressing genes, such as CCK and POMC.

As a key component of the protein digestion cascade, trypsin not only directly participates in substrate hydrolysis but also regulates the entire proteolytic process by activating its zymogen form [31]. This study found that carnivorous fish have a greater advantage in the number of trypsin genes, with species like the bass (a representative of carnivorous groups) undergoing extensive gene duplication, resulting in a higher copy number compared to omnivorous and herbivorous fish. This phenomenon is adapted to the food consumption processes of different species. Over long evolutionary periods, carnivorous fish have enhanced their protein digestion capacity through gene duplication strategies to meet the metabolic demands of a high-protein diet [32]. Similarly, carnivorous perch and herbivorous fish such as grass carp have adapted to their distinct dietary patterns through amplification of the sweet taste receptor T1R2 gene [33]. Furthermore, evolutionary analysis indicates that several digestive enzymes in carnivorous fish have evolved into distinct lineages, suggesting that carnivorous fish have developed new trypsin subtypes to meet their protein digestion needs. However, most fish still retain trypsin isoforms homologous to those in mammals and share similar protein structures. Overall, the increase in digestive enzyme gene copy numbers helps species cope with diverse food consumption demands while potentially adapting to different food preferences and ecological niche distributions [19,34].

Compared to pepsin, trypsin plays a crucial role in the early feeding process of fish larvae by rapidly digesting large amounts of protein [35]. During mandarin fish development (0–48 hpf), trypsin is not expressed initially. However, as the pancreas forms, trypsin begins to be expressed, and its expression gradually extends to the intestines. The precocious expression of trypsin may serve two purposes: one is to facilitate the better intake of yolk nutrients, and the other is to prepare for the digestion of exogenous nutrients. This differs from other digestive enzymes such as pepsin, amylase, and chymotrypsin, which exhibit low expression levels in the early developmental stages [36]. Although the mandarin fish genome contains 13 trypsin genes, only a few of them show high expression during early developmental stages. This pattern may be attributed to differential demands for trypsin isoforms at various growth phases of the fish. Notably, after mandarin fish open their mouths, the expression of trypsin significantly increases compared to before mouth opens. This suggests that trypsin is not maternally inherited and that its expression is programmed early in development to increase significantly at the onset of feeding to facilitate digestion process in mandarin fish larvae. Current research has shown that, when fed a high-protein diet, carnivorous fish significantly increase the expression levels of trypsin, while no such changes occur in herbivorous fish [37]. Additionally, trypsin has been shown to exhibit higher activity in carnivorous fish compared to herbivorous species [37,38]. It is important to note that this change is a result of gene expansion, leading to a dosage effect that increases the overall synthesis of zymogens, thereby promoting the increased expression of digestive enzymes. Furthermore, this may also involve subfunctionalization, which leads to tissue-specific expression differentiation [39]. We note that trypsin is not only expressed in the pancreas but is also abundantly present in the intestines. This phenomenon of heterologous expression has also been observed in turbot (*Scopthalmus maximus*) and zebrafish [40,41]. This may be related to the functional diversity of trypsin, which, in addition to its role as a digestive enzyme, can also act as a signaling factor to regulate various physiological and pathological processes [13]. These processes are mediated by the modulation of signaling pathways such as mTOR and Wnt [42,43]. It is also important to note that trypsin is secreted by pancreatic acinar cells as an inactive zymogen, trypsinogen, which is subsequently activated in the intestinal lumen under the action of enterokinase [44]. This activation mechanism is especially critical during early ontogeny, as the timely functional maturation of the protease system must align with the transition from endogenous (yolk-based) to exogenous (prey-based) nutrition. Delayed or inadequate activation of trypsinogen could significantly impair protein digestion and thus limit larval survival during this nutritional transition.

Since the gene expression of trypsins in mandarin fish during early development (prior to first feeding) is independent of exogenous food intake, it is reasonable to hypothesize that this mechanism may be associated with endocrine-regulated biological rhythms. Appetite factors play a significant role in regulating trypsin expression, whereby appetite-stimulating factors (such as GHRL) and inhibitory factors (such as CCK) interact to achieve a balance in *trypsin* expression [17]. Therefore, the expression of genes that seem to be involved in regulating nutrition molecules in fish larvae (GHRL and CCK) could reveal the transition to exogenous feeding [45,46]. Conversely, trypsin can also feedback-regulate the expression of appetite factors, thereby mediating feeding behavior [19]. We inhibited trypsin activity with a trypsin inhibitor, and the feeding rate of mandarin fish larvae significantly decreased, further suggesting that trypsin may somehow regulate onset of appetite. This is consistent with observations in mice. CCK is an important regulator of enzyme secretion in adult mammals and teleost and it reduces food intake by increasing satiety [47]. After inhibiting trypsin activity, we observed an upregulation of CCK expression, indicating that trypsin can regulate CCK expression to control appetite. Although the change in CCK2 expression was not substantial, only a small amount of CCK is required to alter feeding behavior in both teleosts and mammals. The upregulation of POMC, the most direct appetite suppressor, further supports this view, implying that appetite regulators not only control digestion but may also be inversely regulated by the expression of digestive enzymes. In addition to its classic role as an anorexigenic factor that suppresses appetite, CCK also influences the digestive process itself by regulating intestinal movement and peristalsis. This multifunctional nature highlights the central role of CCK in coordinating digestion and nutrient absorption [48,49]. When trypsin function is impaired, the digestive capacity of mandarin fish larvae is reduced. To prevent indigestion, the expression of CCK is upregulated, which accelerates intestinal motility and shortens digestion time. These findings provide an important insight: enhancing trypsin activity may suppress the expression of appetite-suppressing factors, thereby improving the first feeding rate in mandarin fish. For instance, dietary supplementation of arginine could be applied to enhance trypsin activity, or leucine could be added to activate the mTOR signaling pathway and promote trypsin expression, thus stimulating feeding behavior [42,50]. Furthermore, both trypsin and CCK could serve as potential molecular markers for early selection of superior mandarin fish strains, enabling the identification of individuals with stronger feeding motivation and better digestive adaptability.

## 5. Conclusions

In summary, this study revealed through phylogenetic analysis that the genomes of carnivorous fish have undergone a significant expansion of trypsin genes during evolution, accompanied by the emergence of specific evolutionary clades. This likely represents an adaptive trait developed to meet the physiological demands of efficiently digesting live prey. Furthermore, by investigating the spatiotemporal expression pattern of a representative gene, trypsin-3b, we demonstrated that trypsin expression is not maternally inherited but is programmed early in development. This indicates that trypsin expression is closely associated with carnivorous feeding habits and the specialized feeding behavior at the onset of exogenous feeding. Subsequent inhibitor experiments uncovered a feedback regulatory mechanism between digestive enzymes and appetite-related genes, showing that trypsin modulates feeding behavior by regulating the expression of CCK and POMC. These findings provide new insights into the molecular regulatory network underlying early digestive system development in mandarin fish and offer a valuable reference for genetically improving the adaptation of larval feeds.

## Data Availability

All data used in this study are available in the Supplementary Materials or from NCBI.

## Supplementary Materials

The following supporting information can be downloaded at: https://www.mdpi.com/article/10.3390/cells14191537/s1, Figure S1: Expression profiles of digestive enzymes in early developing mandarin fish; Figure S2: Expression of chymotrypsin under trypsin inhibitor treatment; Figure S3: Spatiotemporal expression pattern of CCK1 and CCK2 in mandarin fish; Table S1: Trypsin gene IDs used in this study; Table S2: Primer sequences for whole-mount in situ hybridization; Table S3: Primer sequences for RT-qPCR.
