# Supplementary material for: Functional Characterization of Trypsin in the Induction of Biologically Live Bait Feeding in Mandarin Fish (Siniperca chuatsi) Larvae
Source: Cells. 2025 Oct 1;14(19):1537. doi: 10.3390/cells14191537 (PMC12523667; doi:10.3390/cells14191537)
Supplement: Supplementary file 1 [file cells-14-01537-s001.zip › Supplementary Materials.pdf]

Supplementary Materials for

# Functional characterization of trypsin in the induction of biologically live bait feeding in mandarin fish (*Siniperca chuatsi*) larvae

**Xiaoru Dong[1,2], Ke Lu[1,2], Jiaqi Wu[1,2], Qiuling Wang[1,2], Xu-fang Liang[1,2,*]**

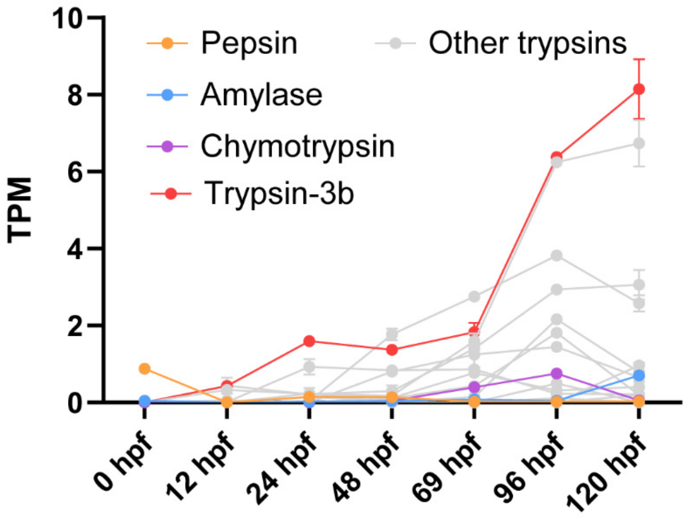


**Figure S1. Expression profiles of digestive enzymes in early developing mandarin fish.** Expression of pepsin, amylase, chymotrypsin, and trypsins from 24 to 120 hpf (TPM).

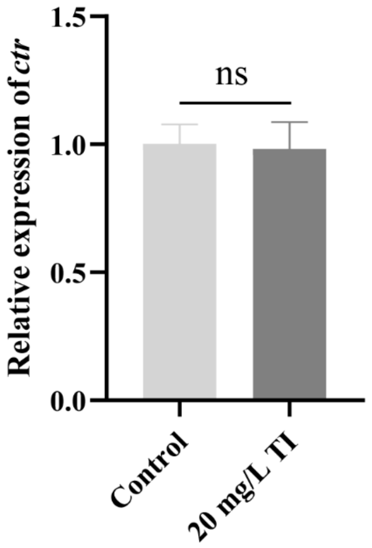


**Figure S2. Expression of chymotrypsin under trypsin inhibitor treatment.** The expression level of chymotrypsin (*ctr*) was detected by qRT-PCR after treatment with trypsin inhibitor at a concentration of 20 mg/L. Culture water was used as the control.

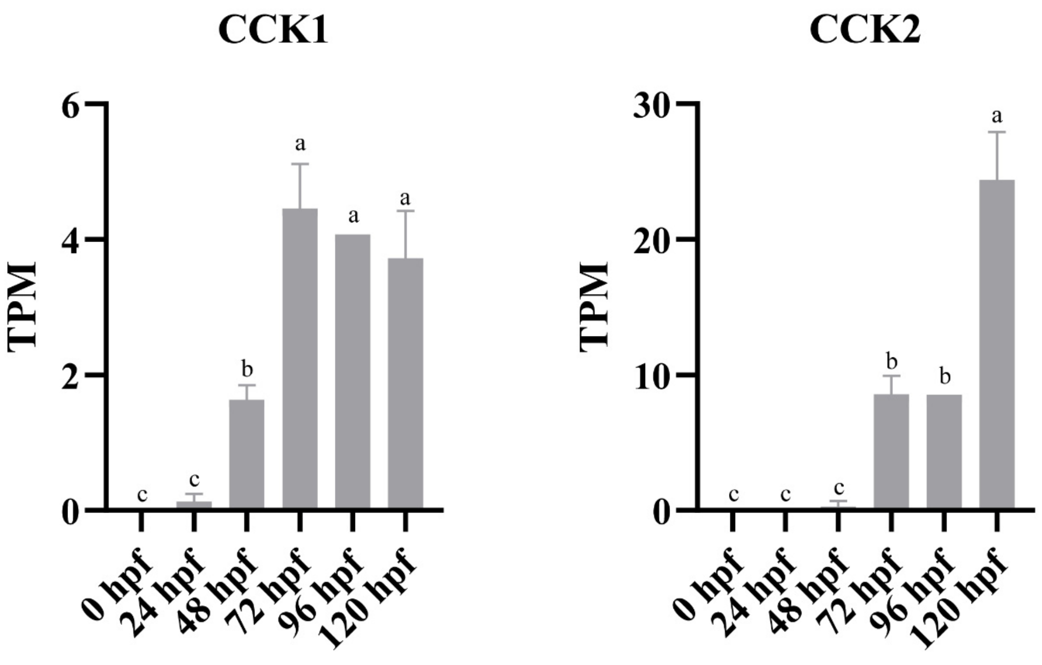


**Figure S3. Spatiotemporal expression pattern of CCK1 and CCK2 in mandarin fish.** Gene expression analysis of CCK1 and CCK2 expression in mandarin fish at 24, 48, 72, 96, and 120 hpf.
